# The Past, Present and Future of Cyber-Physical Systems: A Focus on Models

**DOI:** 10.3390/s150304837

**Published:** 2015-02-26

**Authors:** Edward A. Lee

**Affiliations:** EECS Department, University of California, Berkeley, CA 94720-1770, USA; E-Mail: eal@eecs.berkeley.edu; Tel.: +1-510-643-3728

**Keywords:** cyber-physical systems, real-time systems, clock synchronization, time synchronization, PRET machines, distributed systems

## Abstract

This paper is about better engineering of cyber-physical systems (CPSs) through better models. Deterministic models have historically proven extremely useful and arguably form the kingpin of the industrial revolution and the digital and information technology revolutions. Key deterministic models that have proven successful include differential equations, synchronous digital logic and single-threaded imperative programs. Cyber-physical systems, however, combine these models in such a way that determinism is not preserved. Two projects show that deterministic CPS models with faithful physical realizations are possible and practical. The first project is PRET, which shows that the timing precision of synchronous digital logic can be practically made available at the software level of abstraction. The second project is Ptides (programming temporally-integrated distributed embedded systems), which shows that deterministic models for distributed cyber-physical systems have practical faithful realizations. These projects are existence proofs that deterministic CPS models are possible and practical.

## 1. Introduction

A cyber-physical system (CPS) is an orchestration of computers and physical systems. Embedded computers monitor and control physical processes, usually with feedback loops, where physical processes affect computations and *vice versa*.

Applications of CPS include automotive systems, manufacturing, medical devices, military systems, assisted living, traffic control and safety, process control, power generation and distribution, energy conservation, HVAC (heating, ventilation and air conditioning), aircraft, instrumentation, water management systems, trains, physical security (access control and monitoring), asset management and distributed robotics (telepresence, telemedicine).

As an intellectual challenge, CPS is about the intersection, not the union, of the physical and the cyber. It combines engineering models and methods from mechanical, environmental, civil, electrical, biomedical, chemical, aeronautical and industrial engineering with the models and methods of computer science. This paper argues that these models and methods do not combine easily and that the consequently CPS constitutes a new engineering discipline that demands its own models and methods.

The term “cyber-physical systems” emerged around 2006, when it was coined by Helen Gill at the National Science Foundation in the United States. The related term “cyberspace” is attributed to William Gibson, who used the term in the novel *Neuromancer*, but the roots of the term CPS are older and deeper. It would be more accurate to view the terms “cyberspace” and “cyber-physical systems” as stemming from the same root, “cybernetics,” which was coined by Norbert Wiener [1], an American mathematician who had a huge impact on the development of control systems theory. During World War II, Wiener pioneered technology for the automatic aiming and firing of anti-aircraft guns. Although the mechanisms he used did not involve digital computers, the principles involved are similar to those used today in computer-based feedback control systems. His control logic was effectively a computation, albeit one carried out with analog circuits and mechanical parts, and therefore, cybernetics is the conjunction of physical processes, computation and communication. Wiener derived the term from the Greek ϰυβερνήτης (kybernetes), meaning helmsman, governor, pilot or rudder. The metaphor is apt for control systems.

The term CPS is sometimes confused with “cybersecurity,” which concerns the confidentiality, integrity and availability of data and has no intrinsic connection with physical processes. The term “cybersecurity,” therefore, is about the security of cyberspace and is thus only indirectly connected to cybernetics. CPS certainly involves many challenging security and privacy concerns, but these are by no means the only concerns.

CPS connects strongly to the currently popular terms Internet of Things (IoT), Industry 4.0, the Industrial Internet, Machine-to-Machine (M2M), the Internet of Everything, TSensors (trillion sensors), and the fog (like the cloud, but closer to the ground). All of these reflect a vision of a technology that deeply connects our physical world with our information world. In our view, the term “CPS” is more foundational and durable than all of these, because it does not directly reference either implementation approaches (e.g., the “Internet” in IoT) nor particular applications (e.g., “Industry” in Industry 4.0). It focuses instead on the fundamental intellectual problem of conjoining the engineering traditions of the cyber and the physical worlds. One can talk about a “cyber-physical systems theory” in a manner similar to “linear systems theory”.

Like linear systems theory, a CPS theory is all about models. Models play a central role in all scientific and engineering disciplines. However, since CPS conjoins distinct disciplines, which models should be used? Unfortunately, models that prevail in these distinct disciplines do not combine well, as I explain next.

## 2. Models

The act of modeling involves three distinct concepts: the thing being modeled, the model and the modeling paradigm. For example, a Newtonian model of a mass and a spring (the thing being modeled) consists of an ordinary differential equation (ODE) (the model). The modeling paradigm is the mathematics of calculus and differential equations. Such a Newtonian model might be used by a mechanical engineer to design or analyze a mechanical system.

A very different example is a computer program written in C (the model), which models the behavior of an electrical machine (a computer) that transforms binary data stored in electrical memory. Here, the modeling paradigm is the computer science theory of imperative programs.

Engineers often conflate the model with the thing being modeled. For example, electrical engineers may refer to an ODE as “the system” and use it to assert, for example, that “the system is stable.” Such a statement, however, is not a valid statement about a physical system. It is a statement about a model of the physical system. Arguably, any definitive statement about a system (stability, determinism, timeliness, reliability, safety) is in fact a statement about a model and not a statement about the thing being modeled. I call this idea the “Kopetz principle,” after Hermann Kopetz, from whom I learned it. Emphasizing the need to avoid conflating the model with the thing being modeled, Solomon Wolf Golomb famously stated “you will never strike oil by drilling through the map” [2].

However, this does not, in any way, diminish the value of a map. A model imitates the thing being modeled. A model may serve many purposes. In science, the purpose of a model is generally to yield insight into the thing being modeled and to predict its behavior. In engineering, however, models are often used to design systems that do not yet exist. In this case, the model serves as a specification. It is now incumbent on the physical system to imitate the model, rather than the other way around. A C program, for example, gives the design, and the silicon and wires of the physical realization are expected to imitate the behavior specified by the program.

The value of a model depends on our ability to understand and analyze the model. This ability follows from the choice of modeling paradigm. A poor choice leads to less useful models. For example, we could choose to model the behavior of a computer using differential equations describing the semiconductor physics, but the resulting model will not be understandable or analyzable in the way that a C program is. For example, the resulting model will not easily reveal the mathematical function being calculated by a program.

The value of a model also depends on the model fidelity, the degree to which the model imitates the thing being modeled (or *vice versa*). We can make definitive statements about models, from which we can infer the properties of system realizations. This inference is valid if the model is faithful to the realization (or *vice versa*).

Model fidelity is always approximate, however. Models may be more or less abstract. A more abstract model omits more information about the thing being modeled. A C program, for example, is a more abstract model of what a computer does than differential equations that describe the physics of the electronics. However, both models are modeling the same thing.

It is not always true that a less abstract model is more faithful than a more abstract one. I give examples in [3], which studies discrete physical phenomena, such as collisions between rigid objects, friction and electrical switching. Collisions between rigid objects, for example, involve localized plastic deformation, viscous damping in the material and acoustic wave propagation. Much experimental and theoretical work has been done to refine models of such phenomena, leading to considerable insight into the underlying physical phenomena. However, there is so much uncertainty in the parameters of such details, that much simpler models may be just as effective at predicting the behavior; perhaps even more effective, since the simpler models are more tractable for more complex systems. If the goal is engineering, rather than science, then we are probably more interested in developing insight about macroscopic system behavior than in understanding the physics of collisions, and more abstract models may be more effective.

### 2.1. Deterministic Models

One particularly useful property that models may have is determinism. A deterministic model has exactly one behavior, given the inputs to the model. Such a property is valuable, because it can unambiguously define what the “correct” behavior of the thing being modeled is, given the same inputs. Such a model can be used to develop tests, for example, that determine whether an engineered physical system is sufficiently faithful to the model to be “correct” and, hence, ready to ship. A nondeterministic model is less useful for this purpose, because there are possibly many “correct” behaviors.

While this definition of determinism seems simple, there are considerable subtleties (see [4] for a rigorous treatment). Consider for example a C program. What are the inputs? They might be bit patterns in files or in memory that exist prior to the start of the execution of the program; or they might be data provided during the execution of the program. In the latter case, the time at which those inputs are provided may affect the behavior of the program. Is that time part of what we mean by “the inputs?” If so, then even a simple, single-threaded imperative program may in fact be a nondeterministic model of the behavior of the computer. Such questions need to be clarified in the modeling paradigm before we can sensibly even ask the question of whether the model is deterministic.

A modeling paradigm may be deterministic, meaning that the paradigm only supports deterministic models. Restricting models to such a paradigm has the advantage that models are, by construction, deterministic.

Deterministic modeling paradigms have repeatedly proven extremely useful in science and engineering. One example of such a paradigm is ODEs. Arguably, the determinism of ODEs has played a major role in the industrial revolution and 20th century technology development. Deterministic ODEs can be used to model the dynamics of physical systems and to study their stability and reaction to inputs. For example, civil engineers use such models to predict how structures will behave under loads. Aeronautical engineers use such models to predict how an aircraft will respond to changes in its control surfaces. Mechanical engineers use such models to design moving parts.

A second example of a deterministic modeling paradigm is synchronous digital logic, which abstracts away the random and analog behavior of circuits. Despite intrinsic randomness in the underlying physics (thermal noise, crystal defects, manufacturing variability, diffusion processes, *etc.*), modern chips are extremely faithful to such models. We routinely design circuits that perform the same logical function billions of times per second flawlessly for years. This level of fidelity to deterministic models is unprecedented in human history and arguably provides a critical foundation for the 20th century digital revolution. Analog models are still used for circuits at some interfaces to physical processes (very high frequency systems, such a radio circuits and electromechanical systems, for example). However, even here, the trend has been towards more digital designs (oversampled analog-to-digital converters, for example). The benefits of a deterministic digital abstraction are just too compelling.

The synchronous digital logic modeling paradigm has two valuable and orthogonal parts, “synchronous” and “digital.” Each of these adds value to the models, but it is possible to achieve a deterministic digital abstraction without synchrony. Asynchronous digital circuits allow nondeterministic timing, while preserving deterministic function. At the cost of handshaking logic, such circuits realize well-defined Boolean functions. However, forty years of research in asynchronous digital circuits has failed to yield more than niche applications, despite considerable technical advantages (better electrical noise properties, reduced peak power requirements, higher speed). Synchrony (where a global clock drives latches that record intermediate results) extends determinism into concurrent models in a particularly simple way. Synchronous circuits are easier to understand and analyze.

The core underlying abstractions behind synchronous digital logic have been extended into concurrent software by synchronous languages [5]. Synchronous languages are used in safety-critical embedded systems, such as aircraft control systems. A deterministic model, together with demonstrably faithful implementations, yields designs that are easier to validate and, hence, easier to certify.

A third example of a deterministic modeling paradigm is single-threaded imperative computer programs. The fact that a procedure in such a language implements a deterministic function of its arguments and the state of variables that it accesses is an extremely valuable property. Programmers rely on the determinism of this model to build very large programs. Additionally, the flaws that inevitably arise in large programs are often due to mechanisms that undermine the determinacy of the model, such as threads [6]. Arguably, the determinism of this model provides a critical foundation for the 20th century information technology revolution.

The history of imperative programming languages is instructive. Properties that undermine determinism have been widely viewed as flaws and corrected as languages evolve. For example, the unrestricted use of pointers in C has been tightly constrained in next generation languages, such as Java and C#. The undisciplined use of threads in Java is being corrected today in languages with event-triggered concurrency management, such as JavaScript.

Therefore, here, we have three deterministic modeling paradigms that have had a huge impact on human society. Should we banish nondeterminism in models used for engineering altogether? We examine this question below in Section 2.3. However, first, let us consider the limitations of deterministic models.

### 2.2. Limitations of Deterministic Models

The real world is full of uncertainty. Even a synchronous digital logic implementation in silicon may not behave as predicted by its deterministic model. What if the temperature is too high? What if mechanical stresses damage the silicon? A single-threaded imperative program executed in a physical microprocessor also may not behave as the model predicts. What if an alpha particle flips a bit (this is called a soft error, where an error occurs without damage to the hardware)? What if power is lost? Additionally, a mechanical system will never behave exactly as predicted by an ODE model. The model operates in a mathematical ideal of real numbers and a time continuum. It requires real-valued parameters, which are infinitely precise, and it assumes idealized dynamics, where integration and differentiation are platonic ideals. Nevertheless, such models are useful. Engineers make systems robust by ensuring margins of safety. It is not sufficient, for example, to prove that a model of a feedback control system is stable. One must also prove that it remains stable under reasonable variations in the parameter values.

One valuable use of nondeterministic models, therefore, is to analyze and understand the circumstances that could lead a physical realization to not behave as specified by a deterministic model.

Not all non-idealities of a physical realization, however, are best modeled nondeterministically. Consider a differential equation model being used for software simulation. The parameters are quantized to be representable in a finite number of binary digits. Hence, although, in principle, the precision of a parameter is infinite, its accuracy is not. For example, even if we know from the principles of physics that a parameter of a model has value *π*, exactly, but that parameter gets represented in a standard double-precision floating point number, then its representation will not be exact. In addition, a software simulation model will suffer from inaccuracies if the behavior is determined by numerical integration (*vs.* symbolic solvers). Rather than model these non-idealities nondeterministically, a software simulation of a differential equation model could instead be viewed as a different model. It is not the same model as the ODE, a platonic ideal. The fidelity of the simulation model to the physical system will depend on the numerical integration technique that is used. However, a well-designed modeling tool will still be providing a deterministic modeling paradigm. Just because numerical integration is being used does not mean that the results of running a simulation are not a function of the inputs provided.

Another limitation of deterministic models is complexity. Nondeterministic models can efficiently model entire families of behaviors, whereas a deterministic model models each behavior individually. Deterministic models may therefore be much bigger than comparable nondeterministic models (more states, more modeling elements, *etc.*). A well-known result in automata theory is that every nondeterministic Finite-State Machine (FSM) has a deterministic equivalent. However, the deterministic equivalent tends to have many more states. Smaller, more abstract nondeterministic models may be easier to understand and often yield to formal analysis more readily than larger, deterministic models. Model checking [7], for example, easily handles nondeterministic models, and since the tractability of the algorithms depends heavily on the size of the model, a property (such as a safety property) may be verifiable on a nondeterministic model and not (practically) verifiable on a comparable deterministic model.

Finally, nondeterministic models are the only reasonable option when unknown or unknowable properties are central to the model. The behavior of traffic over the Internet depends on unknown (and, in practice, unknowable) human behavior. Models of extremely small physical systems, for example, may come up against quantum mechanical uncertainty principles.

Even the best deterministic modeling paradigms have limitations. Consider the problem of predicting the behavior of multiple simultaneous collisions of rigid objects, for example billiard balls. Newton's laws provide simple ways to model the motion as ODEs, but the collisions are not so simple. In [3], I study how to construct simple deterministic models of such collisions, and I show that such deterministic models are incomplete. Consider a one-dimensional example with three billiard balls, one of which is stationary, while the other two approach it from opposite sides. Let *τ* be the time between the first collision and the second. As long as *τ* ≠ 0, it is easy to construct a deterministic model that obeys Newton's conservation laws and laws of motion. However, when *τ* = 0, if the masses of the balls are not the same, then more than one solution satisfies Newton's laws. Consider a sequence of distinct models where *τ* approaches zero; e.g., let *τ* take values from the sequence (1, 1/2, 1/4, 1/8, … ). Each value of *τ* corresponds to a different model, so this sequence gives us a sequence of models. Every model in the sequence is deterministic (there is exactly one solution that satisfies Newton's laws). Moreover, the sequence has a well-defined limit, a model that predicts a single unique behavior when *τ* = 0. However, if instead, we take *τ* from the sequence (−1, −1/2, −1/4, −1/8, … ), then the limit model is again a well-defined deterministic model, but it is not the same model. A reasonable interpretation is that when *τ* = 0, the model becomes nondeterministic, and either of the two limiting behaviors is allowed. This interpretation appears to be defensible based on the underlying physics, where the notion of “simultaneous collisions” requires deeper study using relativity and quantum mechanics.

In [3], I relate this incompleteness of determinism to the notion of constructive semantics, introduced by Berry [8]. Berry applied the concept to synchronous languages and later to synchronous circuits [9]. Loosely, a program or circuit is constructive if a simple procedure that iteratively completes incomplete information terminates and yields a behavior for the program or circuit. In synchronous languages and digital circuit models, it is easy to ensure that this procedure always terminates.

The notion of constructiveness now appears to apply, as well, to continuous dynamics [3]. As long as *τ* ≠ 0 in the above billiard ball models, the models are constructive. However, when *τ* = 0, the model becomes nonconstructive. Additionally, in this case, nondeterminism appears to be a reasonable way to deal with nonconstructive models.

### 2.3. Uncertainty, Probability and Nondeterminism

Given the limitations of deterministic models, should we abandon them altogether, despite their considerable value? The answer is a qualified “no”. We should not abandon them unless they are truly incapable of accomplishing what we need.

Suppose, for example, that we are trying to understand how a single-threaded imperative program will be affected by an alpha particle that causes a soft error? These events are rare, and each event will typically affect only one of billions of bits that are used in the design. Determining the possible consequences of such an event can be extremely difficult. One approach would be to modify the deterministic modeling paradigm, replacing it with a nondeterministic one that assumes that between any two operations of a single-threaded imperative program, the state of the memory can change arbitrarily. This new modeling paradigm, however, would not be very useful. This modified modeling paradigm could also apply to multithreaded programs, where it results in equally useless models [6]. Nondeterminism is clearly needed in this scenario, but we do not need to go such extremes.

Another approach would modify the modeling paradigm to admit probabilistic behaviors. For example, we could assume that between any two operations of a single-threaded imperative program, the state of each bit in memory can change with a very small probability. This is not the same as nondeterminism. Nondeterminism is about possibility (whether it is possible, within a modeling paradigm, for something to occur), whereas probability is about likelihood.

There is a long-standing philosophical debate where statisticians interpret probabilistic models fundamentally differently. The so-called “frequentists” interpret a probability as representing the fraction of times that one outcome is observed *vs*. another in a large number of repeated experiments. The so-called “Bayesians” interpret a probability as representing the uncertainly that humans (those building the models) have about the thing being modeled. The choice of interpretation gives us the intellectual framework within which to assess the fidelity of the model.

The frequentist interpretation seems reasonable when the thing being modeled actually has repeated experiments, for example in a model of car accidents that occur in millions of actual car trips. It does not make much sense, however, if the thing being modeled has no (or few) repeated experiments, for example a model of the landing of a spacecraft on an asteroid. Although both interpretations may lead to the same models, the Bayesian interpretation has the advantage that it provides a systematic way to incorporate prior knowledge or biases about the system. More importantly, it provides a way to use online observations to improve the model, as done by many machine learning algorithms, such as particle filtering and Bayesian networks.

Nondeterministic and probabilistic models are useful and necessary for systematically handling unknown or unknowable events and behaviors. Even though there are always unknown and unknowable aspects, this does not undermine the value of deterministic models. The Golomb principle (do not conflate the model with the thing being modeled) means that a deterministic model does not become useless just because the thing being modeled will behave differently.

Use of deterministic models does not eliminate the need for fault-tolerant design. Quite the contrary, a deterministic model clarifies what a “fault” is (any deviation from the model) and, hence, makes it easier to build-in fault tolerance.

## 3. Modeling Cyber-Physical Systems

Deterministic models are valuable. They form the foundation of every engineering discipline. Differential equations model the physical dynamics of electrical, mechanical and many other systems. Synchronous digital logic models electrical circuits and, more recently, concurrent software. Imperative programs model computations performed in a computer.

Cyber-physical systems combine physical dynamics with computational processes, so it would seem that they could leverage the power of these deterministic models. Unfortunately, combinations of these deterministic models for physical dynamics and for computation are nearly always nondeterministic. The principal reason for this is the lack of temporal semantics in programs.

### 3.1. Controlling Timing in Programs

**Figure 1.**
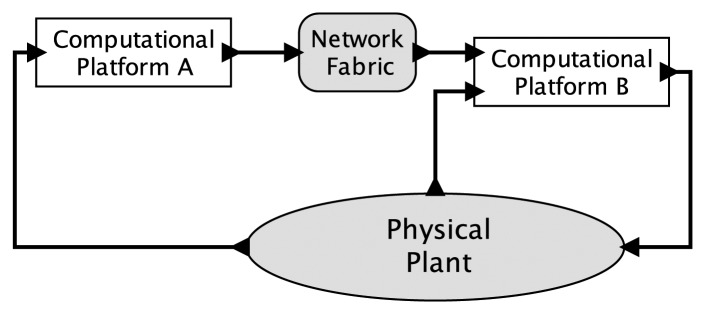
Structure of a simple cyber-physical system (CPS).

Consider a simple CPS depicted in Figure 1. Here, two embedded computers interact with a physical plant through sensors and actuators and with each other through a network fabric. Suppose that the physical plant is modeled with an ODE and that the computations are modeled with single-threaded imperative programs. Each of these models is deterministic, but the combination is not. The behavior of an ODE is strongly affected by the timing of its external stimuli, but a program provides at best only very weak ways to control that timing. Worse, even those weak mechanisms are rarely part of the semantics of the programming language, so the program, viewed as a model, actually expresses nothing at all about timing.

Consider, for example, a program written in C, the most widely-used language for CPS implementations today The C language provides no mechanisms to express timing behavior. To control timing, the programmer has to step outside the modeling paradigm of the C language, for example by writing bit patterns to a memory-mapped register to trigger a timer interrupt. Within the framework of the C language, this action merely writes a bit pattern to a memory location. Nothing more. The side effect of triggering a timer interrupt is not expressed at all in the program and, in fact, will depend on the particular hardware on which the program is run. The model (the program) and the thing being modeled (the hardware on which it runs) get conflated, because the intended behavior is not expressed in the model, but rather emerges from the implementation.

The reader may protest at this point that the computations may be performed under the control of a real-time operating system (RTOS) [10] and that the capabilities provided by the RTOS should be considered part of the model. This is valid, but the most-widely used RTOS mechanisms fall short of yielding a deterministic modeling paradigm. In a deterministic modeling paradigm, behavior that deviates from the model is a fault. However, RTOSs rely primarily on priorities, and any execution that obeys the priorities is deemed “correct”. Typically, there will be many such behaviors, exhibiting many possible interactions with the physical plant.

There is a long history of programming languages that include timing constructs (Modula [11,12], PEARL (Process and Experiment Automation Realtime Language) [13], Ada [14], Occam [15], Real-Time Euclid [16] and Erlang [17], for example). These improve things by including in the language some of the mechanisms of an RTOS, which means that a model (a program) is more self-contained. One does not need to combine the semantics of the language with the semantics of a separate and distinct RTOS to interpret the model. These languages, however, also fall far short of yielding a deterministic modeling paradigm. This is, in part, for very practical reasons. The underlying computers do not provide in their instruction set architectures mechanisms for controlling timing, and in fact, the trend has been towards less predictable and controllable timing (see Section 4 below). Either the timing specified by programs in such languages will be coarsely approximated by the physical implementation or the physical implementation will have to be over-provisioned, so that the variability it exhibits in timing is sufficiently small.

There are two ways to improve this situation. First, we can improve the timing determinism of the underlying computers. I examine this approach in Section 4 below. Second, we can change the notion of time in programs, so that it has a logical correctness irrespective of the timing of the underlying computers. I examine this approach in Sections 5 and 6.

### 3.2. The Cost of the Missing Temporal Semantics

Although the weaknesses of real-time mechanisms have been known for some time [18], little progress has been made. Today, real-time systems require considerable over-provisioning if the timing is important. This increases cost and energy consumption and decreases the reliability by increasing the part count.

Perhaps even more importantly, because timing emerges from the implementation, validating the models is not very useful. Engineers are forced instead to validate the implementation. This results in an implement-and-test style of design that dominates embedded systems today. It is hacking, not based on sound engineering principles.

One rather dramatic consequence of these failings is that manufacturers of cyber-physical systems cannot easily replace or update the hardware that is used to execute embedded software. An extreme example of this is found in commercial aircraft. These are safety-critical systems with extensive (and expensive) certification requirements. In a fly-by-wire aircraft, the flight control system is realized in software, but it is not enough to certify the software. As a consequence, manufacturers of aircraft (such as Boeing and Airbus) stockpile enough microprocessors to last for the entire production run of a plane. The production run for a typical commercial airplane is, perhaps, 50 years, so towards the end of the production run, the plane will be made with microprocessors that are 50 years old!

What gets certified is the implementation, not the model. Indeed, with today's models, it has to be that way. It is not enough to correctly execute the C code that implements the flight control system. Two distinct microprocessors that execute the code correctly will exhibit different timing, and therefore, the behavior of the cyber-physical composition will be different. Replacing a microprocessor with a more modern one would require repeating the (expensive) certification process.

To emphasize what I mean by “correctly” executing a program, consider the following C procedure:
void foo ( int 32_t x) { if ( x > 1000 ) {  x = 1000; } if ( x > 0 ) {  x = x + 1000;  if ( x < 0 ) {   panic ( ) ;  } }}

In C, we need to make some assumptions in order to infer the deterministic behavior of the above procedure. Specifically, we need to assume that no other thread or interrupt-service routine can overwrite the stack or the program memory. In more modern languages, such assurance is provided by construction, as part of the language definition. With these assumptions, however, the above procedure is deterministic, in that given a 32-bit integer argument, it will exhibit exactly one behavior.

Furthermore, the one behavior does not include invocation of the panic routine. No correct execution of this procedure will invoke it. A soft error or a violation of the above assumptions could result in invoking panic, but those would not be correct executions. Viewed as a model, we can develop absolute confidence in the software, in that only a hardware failure is an excuse for invoking panic. No such assurances are available for timing properties, however.

With respect to the invocation of the panic procedure, the program above is an extremely high fidelity model. Today's microprocessors execute such programs with astonishing reliability. Faults are rare. We can credit the power of another deterministic modeling paradigm, synchronous digital logic, for this fact. However, interestingly, synchronous digital logic is also capable of extremely precise and repeatable timing. It is by choice that this capability is not exposed in software abstractions. Software abstractions were not created for cyber-physical systems. They were created to run payrolls.

Some aircraft designers (notably Airbus) use synchronous languages, not just C, in their flight control systems. This improves the situation by making the software concurrency model deterministic by construction. However, it does not improve the situation enough. Correct execution of a synchronous program does not, in fact, depend on how long it takes to do anything. It only depends on coordination between the concurrent components. If one gets delayed, the others may need to be delayed, too. The Giotto programming model [19] improves things by introducing the notion of logical execution time. However, Giotto has not been widely adopted. Giotto can be viewed as an elevation of the concept of a time-triggered architecture [20] in the programming model.

More commonly, system vendors use C for their safety-critical software. Even with C, deterministic concurrency is possible using a stylized design. For example, designers could avoid threads and interrupt-driven I/O and write programs where tasks execute in fixed, round-robin schedules. In this case, although determinism is not assured by construction, it is (hopefully) assured by policy and code review, but at considerable cost to efficiency. Handlers for rare or sporadic events, for example, will consume fixed resources even when nothing is happening. Although this can yield high-confidence designs, the programming model still leaves out timing, so the behavior of the cyber-physical combination still emerges from the implementation rather than being given in a model. For example, the round-robin schedule might be synchronized with a hardware implementation of a time-triggered communication bus.

Vendors that are not subject to stringent certification requirements may not even attempt to ensure deterministic concurrency. This can be extremely risky, as evidenced by the considerable criticism of Toyota's software that resulted from investigations of possible cases of unintended acceleration [21]. The design style in that software extensively uses unguarded shared variables accessed by concurrent threads. As a result, the programs have many correct behaviors, and given the same inputs, there are too many to test. Although there is no conclusive evidence that flaws in the software caused any unintended acceleration events, Toyota nevertheless suffered searing criticism of their design style [22].

### 3.3. Reconciling Deterministic Models with a Nondeterministic World

Considering Figure 1, there are many sources of nondeterminism in a physical realization: physical noise, part failures, imperfect actuation, packet losses, unknowable delays, unknowable execution times and uncontrollable scheduling, to name a few. In the face of such nondeterminism, does it make sense to talk about deterministic models for cyber-physical systems? I contend that it does. Is this a throwback to Laplace's view of a deterministic universe, which has been thoroughly debunked by modern physics? No, this is about models, not about the physical world. Deterministic CPS models for which there exist high fidelity implementations are possible, and deterministic models have proven so useful in the past, that I expect they will prove useful again.

I explain how next. There are two sides to this story. On the physical implementation side, we can modify the design of computers to gain better control over timing. On the modeling side, we can use better models of time than the Newtonian ideal of a shared universal absolute time. This is a “meet in the middle” approach. Better models together with better implementations yield determinism (on the modeling side) and high fidelity (in the physical implementation).

First, I explain how precise and repeatable timing, which is readily available in synchronous digital logic, can be made available in the software abstractions. This requires rethinking how microprocessors are designed. Second, I explain how distributed software communicating over networks can be given a deterministic timed model of computation for which high-fidelity implementations are possible.

## 4. PRET Machines

Consider a program that wishes to control timing. Suppose, for example, that we need a program to react to an external stimulus (perhaps generated by an interrupt) by writing a control value to a memory-mapped register 42 ms later. First, I note that this requirement is ill posed, because what does “42 ms later” mean? No physical realization can deliver exactly 42 ms later (whatever that means). Any physical manifestation of a timing specification will require tolerances and a timing reference.

Therefore, let us modify the requirement. The program needs to write to a memory-mapped register 42,000 clock cycles later. This requirement is conceptually similar to the previous one, if the clock has a period of one microsecond, but now, the requirement is well posed, if we assume an underlying synchronous digital logic model. This requirement now has a finite tolerance of one microsecond (one clock period). This may introduce nondeterminism in its interaction with the physical world, if our models of the physical world (e.g., ODEs) are still based on a real-valued idealized Newtonian time. However, if we modify our physical models also to have a tolerance, it becomes possible to construct a conjoined deterministic model.

However, the requirement is still problematic, because it is not expressed at the software level of abstraction. There is no way to write a C program that meets this requirement. It might be possible to write a C program that, when executed on a particular piece of hardware, does meet the requirement. However, this conflates the model with the thing being modeled. Additionally, even then, doing so will be difficult with today's microprocessors. Controlling timing at the granularity of clock cycles is extremely difficult. The only way we would know to do this today would be to use a very old microprocessor, where every instruction takes exactly one cycle to execute. In addition, we would need to write the program by hand in assembly language (not in C) and count cycles.

Therefore, this requirement is not good either. Therefore, let us modify the requirement again: the program needs to write to a memory-mapped register 42,000 clock cycles later, plus or minus 100 cycles. We now have a coarser tolerance, which may make it easier to make a C-based model and a faithful realization.

However, it will still not be easy. Analyzing C programs for execution time is extremely difficult; it requires detailed information about the hardware realization, and it requires making assumptions that are often unrealistic [23]. For example, we may have to assume that interrupts are disabled for the entire 42,000 clock cycles, making the system unresponsive for quite a long time. Additionally, again, our model of the physical world will have to work with matching tolerances for the conjoined model to be deterministic.

Nevertheless, with coarse enough tolerances for timing, we can make deterministic CPS models today for such scenarios. However, the cost of doing so is enormous. In practice, in safety-critical systems, draconian measures are taken, such as disabling interrupts and caches, forbidding multitasking and using microprocessors that are orders of magnitude more performant than what is really needed. Additionally, even then, the achievable tolerances are quite coarse compared to what the underlying synchronous digital logic is capable of. This is, put simply, sloppy engineering.

An alternative real-time requirement, very commonly used, is that the program should respond within 42 ms. This imposes a deadline. It assumes that an early response is just as good as one that barely meets the deadline. This requirement is relatively easy to achieve by over-provisioning the physical realization. However, the more timing variability in the physical realization (due to pipelining, caches, memory latencies, interrupts, multitasking, *etc.*), the more headroom that the physical realization needs. Additionally, now, a deterministic CPS model becomes nearly impossible, because “correct” execution of the program (meeting the deadline) can exhibit quite a wide range of behaviors.

Yet another approach to real-time requirements is soft deadlines. The desired response is within 42 ms, but please respond as soon as you can. Such requirements can be systematized using time-utility functions (TUFs) [24], but now, we have essentially given up completely on a deterministic CPS model.

Deadlines, whether hard or soft, suffer from a practical problem that controlling the order of events becomes difficult. If task *A* has a deadline of 21 ms and task *B* has a deadline of 42 ms, there is no assurance (in the model) that *A* will complete before *B*. An implementation may provide such assurance, for example by using a deadline-monotonic scheduling policy [10], but even then, such assurances become increasingly difficult with multicore processors and distributed systems. Changing the order of events can have much more dramatic effects on a physical system than perturbing the timing. Consider, for example, if task A turns on a motor and task *B* turns off the same motor. The order in which these occur yields drastically different behaviors.

We can do better. This section reviews work that demonstrates that it is possible to expose, at the software level, timing tolerances much closer to those of the underlying synchronous digital logic. This greatly facilitates faithful realizations of deterministic CPS models. Timing can be made more precise (lower tolerances) at much lower cost, with lower energy requirements and with higher reliability.

### 4.1. Instruction Set Architectures

A microprocessor executes a sequence of instructions from an instruction set. Each instruction in the instruction set changes the state of the processor in a well-defined way. The microprocessor provides a strong guarantee about its behavior: if you insert in the sequence an instruction that observes the state of the processor (e.g., the contents of a register or memory), then that instruction observes a state equivalent to one produced by a sequential execution of exactly every instruction that preceded it in the sequence. This is a deterministic model, codified in an instruction set architecture (ISA).

The model says nothing about timing, however. A faithful implementation of the ISA is only required to satisfy (with high probability) this strong guarantee. Modern microprocessors include extremely clever techniques for getting high performance while satisfying the guarantee. For instance, they do not typically execute the instructions strictly in sequence. Instead, pipelines, caches, write buffers and out-of-order execution reorder and overlap operations while preserving the illusion of sequential execution.

### 4.2. Precision-Timed ISAs

The PRET project at Berkeley set out in 2007 to demonstrate that an ISA that gives programmers a rigorous timing model does not need to sacrifice performance [25,26]. The acronym “PRET” stands variously for Precision-Timed processors, Predictable, Repeatable Timing and Performance with Repeatable Timing.

The first concrete demonstration of this was the PTARM (Precision Timed ARM) [27], a soft core implemented on an FPGA based on an ARM ISA. PTARM uses a thread-interleaved pipeline to mitigate pipelining effects and an interleaved memory controller [28] to get deterministic latency from a memory hierarchy. The size of the design is nearly the same as comparable 32-bit microprocessors, so there is no particular area penalty to providing programmers with precise control over timing. However, more importantly, the aggregate performance (total useful work done per unit time) actually exceeds that of a comparable conventional pipelined processor, provided that there is enough concurrency in the application to keep four threads busy at all times.

This latter requirement is mitigated by the latest PRET design, called FlexPRET [29], which provides a graduated architecture with flexible scheduling of the pipeline. It supports mixed-criticality systems [30] in that it can combine threads with ironclad timing behavior with threads that opportunistically use any remaining cycles. This architecture is based on the open-source RISC-V [31], is implemented using Chisel [32] and is deployed as an FPGA soft core and a C++ cycle-accurate simulator.

At a high level, PRET machines combine several ideas, few of which are actually new, to give programmers control over timing. First, they use a thread-interleaved pipeline to enable deterministic timing in the pipeline. Second, they leverage the parallelism in DRAM memory designs to interleave accesses, so as to enable deterministic latencies. In FlexPRET, use of these deterministic mechanisms is optional and is presumably done only for those threads for which deterministic timing is essential. Third, management of the memory hierarchy (what data resides where) is done in software rather than in hardware cache controllers [33]. Fourth, interrupt-driven I/O is temporally isolated, so that hard real-time threads are either unaffected by interrupts or the effect on their timing has a rigorous bound. Finally, the ISA is extended with instructions that explicitly control timing (see [34]).

PRET machines bring a number of benefits. They enable programs with repeatable timing, which improves assurance that a deployed system will behave in the field as it did on the test bench. They improve testability, because fewer timing variations need to be considered. They reduce the need for over-provisioning, because timing requirements are explicit in the programs, and it can be statically determined whether a particular physical realization will meet those requirements; this reduces the system cost and may dramatically reduce energy consumption, enabling, for example, much slower clocking of systems when such slower clocking demonstrably meets the timing requirements. They enable replacement of hardware without requiring recertification of the software; if the behavior of the interactions between the software and the physical plant is demonstrably identical when the same program is executed on new hardware, then it becomes possible to certify the software instead of certifying the implementation.

### 4.3. Deploying PRET in Systems

Given these substantial benefits, I predict that PRET machines will become widespread in CPS applications. They may, however, also become widespread in general-purpose computing, which also benefits from improved determinism. Exploiting the parallelism of multicore machines, for example, becomes much easier and more efficient with controllable timing [35]. Even more interestingly, PRET machines might make useful “real-time unit” (RTU) companions to general-purpose computers, in a manner similar to how “graphics processing units” (GPUs) have become ubiquitous. RTUs could handle high-performance I/O and networking protocols, for example. They would be much more flexible than today's hardware implementations of the same. Additionally, their timing capabilities could be exposed to application programs through an “OpenRT” API, analogous to the OpenGL API widely used by GPUs.

As a model, a program that uses the extended ISA of a PRET machine controls timing at a granularity of a clock cycle, thus exposing, at the software abstraction level, the timing precision provided by the underlying synchronous digital logic. For a simple CPS, this provides a deterministic model for the interaction between one PRET machine and one physical plant, provided that the physical plant model uses the same model of time. However, considering the only slightly more elaborate CPS sketched in Figure 1, this timing model falls short of delivering determinism. In particular, suppose the two “computational platforms” in the figure are PRET machines with independent clocks. Those clocks will drift with respect to one another in unpredictable ways. This means that the interactions between those PRET machines and the physical plant have no clean semantic notion of simultaneity, nor even of relative ordering.

One possibility is to drive all PRET machines in a CPS from the same clock. To provide a deterministic model of time, in fact, we must define a modeling paradigm where some notion of time is globally shared. The question then becomes how to provide a faithful physical realization of this modeling paradigm. A brute force technique using a global clock distribution network will suffer from clock skew and, hence, will not be able to operate at anywhere near the granularity of a clock cycle of a single PRET machine. In fact, time-triggered architectures [20] classically use such a clock distribution network, limiting their scale and timing precision. However, more modern techniques rely instead on clock synchronization protocols. I discuss this next.

## 5. Models of Time

Deterministic CPS models will need a semantic notion of time that can be used to model both physical dynamics and temporal behavior in software. It is naive to assume that we can just use the Newtonian ideal, where time is absolute, a real number t, visible everywhere and advancing uniformly. I begin in this section by giving a set of requirements that any useful model of time must satisfy. I then elaborate with desirable (but not required) features and conclude with a description of a practical realization of a model of time that meets all of the requirements and delivers all of the desirable features.

### 5.1. Requirements

Like the Newtonian ideal, any useful semantic notion of time has to provide a clear ordering of events. Specifically, each component in a system must be able to distinguish past, present and future. The state of a component at a “present” is a summary of the past, and it contains everything the component needs to react to further stimulus in the future. A component changes state as time advances, and every observer of this component should see state changes in the same order.

We also require a semantic notion of time to respect an intuitive notion of causality. If one event *A* causes another *B*, then every observer should see *A* ordered before *B*.

We also require a semantic notion of simultaneity. Under such a notion, two events are simultaneous if all observers see them occurring at the same time. We may also want to avoid models where one observer deems two events to be simultaneous and another does not.

I could easily now digress into philosophy or modern physics. For example, how could a notion of simultaneity be justifiable, given relativity and the uncertainty principles of quantum mechanics? How useful is the notion of simultaneity in the physical world? I resist the temptation to digress and appeal instead to practicality. We need models that are useful for engineering. The goal is to be able to design and build better cyber-physical systems, not to unlock the secrets of the Universe. Any model that includes time values faces the possibility that two time values will be equal. The modeling paradigm, therefore, needs to either deal cleanly with the equality of two time values (defining what equality means and defining ordering with near equality) or disallow equality. It could disallow equality using, for example, some physics-inspired uncertainty principle, but such a modeling paradigm would forgo determinism, at potentially great cost. Even after the development of relativity and quantum mechanics, Newtonian ideal time remains a practical choice for studying many macroscopic systems.

However, ironically, Newtonian time proves not so practical for cyber-physical systems. The most obvious reason is that digital computers do not work with real numbers. Computer programs typically approximate real numbers using floating-point numbers, which can create problems. For example, real numbers can be compared for equality (e.g., to define “simultaneity”), but it rarely makes sense to do so for floating point numbers. In fact, some software bug finders, such as Coverity, report equality tests of floating point numbers as bugs.

Consider a CPS where two components (cyber or physical) produce periodic events with the same period starting at the same time. The modeling paradigm should assure that those events will appear simultaneously at any other component that observes them. Without such a notion of simultaneity, then the order of these events will be arbitrary, and as we have observed above, changing the order of events can have a much bigger effect than perturbing their timing. Periods that are simple multiples of one another should also yield simultaneous events. Quantization errors should not be permitted to weaken this property.

Broman *et al.* [36] list three requirements for a model of time:

1. The precision with which time is represented should be finite and should be the same for all observers in a model. Infinite precision (as provided by real numbers) is not practically realizable in computers, and if precisions differ for different observers, then the different observers will not agree on which events are simultaneous.
2. The precision with which time is represented should be independent of the absolute magnitude of the time. In other words, the time origin (the choice for the meaning of time zero) should not affect the precision.
3. The addition of time should be associative. That is, for any three time intervals *t*_1_, *t*_2_ and *t*_3_,
  $$\left(t_{1}+t_{2}\right)+t_{3}=t_{1}+\left(t_{2}+t_{3}\right).$$

The last two of these three properties are not satisfied by floating-point numbers, so floating-point numbers should not be used as the primary representation for time. The first property implies that the precision of the representation of time should be a global property of a model, not a property of individual components in the model.

I add to this a fourth requirement:

4. Monotonicity: any observer of time in a model that is a sequential process (a sequence of state changes) should observe non-decreasing values of time.

We also do not need for time to advance uniformly throughout a model, as long as ordering and simultaneity are preserved where needed. I examine this issue next.

### 5.2. Hierarchical and Multiform Time

Useful models are usually hierarchical, where a component at one level of the hierarchy is actually a composition of subcomponents at a lower level of the hierarchy. In such models, it is not necessary for time to advance uniformly at all levels of the hierarchy. The hierarchy itself provides a convenient mechanism for preserving ordering and simultaneity without requiring uniformity.

Modal models [37] benefit particularly from a hierarchical notion of time. In a modal model, a component at one level of the hierarchy has one or more modes. Each mode represents a distinct behavior. For example, an object on a surface may have two modes, sliding, where the object is sliding on the surface, and resting, where it is not. The two modes may be described by distinct submodels. Each submodel is active only some of the time. During the time it is inactive, what should the submodel's view of time be doing? Lee and Tripakis [37] argue that local time should freeze while environment time advances. They give a semantics that preserves causal ordering, determinism and simultaneity.

Freezing time in the inactive mode of a modal model is one form of hierarchical time. Another form that proves useful allows time to progress at different rates at different levels of the hierarchy [38]. This feature is particularly useful for modeling distributed systems where maintaining a perfectly coherent uniform time base is not physically possible. It is referred to as multiform time, and it enables highly realistic models that explicitly recognize that time can only be imperfectly measured.

### 5.3. Superdense Time

A useful variant of a model of time is superdense time [39–42]. A superdense time value is a pair (*t*, *n*), called a time stamp, where *t* is the model time and *n* is a microstep (also called an index). The model time represents the time at which some event occurs, and the microstep represents the sequencing of events that occur at the same model time. Two time stamps (*t*, *n*_1_) and (*t*, *n*_2_) can be interpreted as being simultaneous (in a weak sense), even if *n*_1_ ≠ *n*_2_. A stronger notion of simultaneity would require the time stamps to be equal (both in the model time and microstep).

An example illustrates the value of superdense time (see [3] for many more examples). Consider Newton's cradle, a toy with five steel balls suspended by strings. If you lift the first ball and release it, it strikes the second ball, which does not move. Instead, the fifth ball reacts by rising.

Consider the momentum *p* of the second ball as a function of time. The second ball does not move, so its momentum must be everywhere zero. However, the momentum of the first ball is somehow transferred to the fifth ball, passing through the second ball. Therefore, the momentum cannot be always zero.

Let ℝ represent the real numbers. Let *p*: ℝ → ℝ be a function that represents the momentum of this second ball, and let *τ* be the time of the collision. Then:

(1) $$p(t)=\{\begin{array}{ll} P & \text{if}\;t=\tau \\ 0 & \text{otherwise} \end{array}$$

for some constant *P* and for all *t* ∈ ℝ. Before and after the instant of time *τ*, the momentum of the ball is zero, but at time *τ*, it is not zero. Momentum is proportional to velocity, so:

p(t)=Mυ(t),

where *M* is the mass of the ball. Hence, combining with Equation (1),

(2) $$\upsilon (t)=\{\begin{array}{ll} P/M & \text{if}\;t=\tau \\ 0 & \text{otherwise} \end{array}$$

The position of a mass is the integral of its velocity,

$$x(t)=x(0)+\int_{0}^{t}\upsilon (\tau )d\tau ,$$

where *x*(0) is the initial position. The integral of the function given by Equation (2) is zero at all *t*, so the ball does not move, despite having a non-zero momentum at an instant.

The above physical model mostly works to describe the physics, but it has two flaws. First, it violates the basic physical principle of the conservation of momentum. At the time of the collision, all three middle balls will simultaneously have non-zero momentum, so seemingly, aggregate momentum has magically increased. This is a flaw in the notion of simultaneity in the model. Second, the model cannot be directly converted into a discrete representation, making it difficult for computers to operate on the model numerically.

A discrete representation of a signal is a sequence of values that are ordered in time. Any such representation of the momentum in Equation ( 1) or velocity in Equation (2) is ambiguous. If the sequence does not include the value at the time of the collision, then the representation does not capture the fact that momentum is transferred through the ball. If the representation does include the value at the time of the collision, then the representation is indistinguishable from a representation of a signal that has a non-zero momentum over some interval of time and, therefore, models a ball that does move. In such a discrete representation, there is no semantic distinction between an instantaneous event and a rapidly varying continuous event.

Superdense time solves both problems. Specifically, the momentum of the second ball can be unambiguously represented by a sequence of samples where *p*(*τ*, 0) = 0, *p*(*τ*, 1) = *P* and *p*(*τ*, 2) = 0, where *τ* is the time of the collision. The third ball has non-zero momentum only at superdense time (*τ*, 2). At the time of the collision, each ball first has zero momentum, then non-zero, then zero again, all in an instant. The event of having non-zero momentum is weakly simultaneous for all three middle balls, but not strongly simultaneous. Momentum is conserved. Moreover, the model has an unambiguous discrete representation. A sampling that includes three samples at time *τ*, one with value zero, followed by one with a non-zero value, followed by a third with value zero, unambiguously models the collision as a discrete, instantaneous event with zero time duration.

One could argue that the physical system is not actually discrete. Even well-made steel balls will compress; so, the collision is actually a continuous process, not a discrete event. This is true, but when building models, we do not want the modeling formalism to force us to construct models that are more detailed than is appropriate. Such a model of Newton's cradle would be far more sophisticated, and the resulting non-linear dynamics would be far more difficult to analyze. The fidelity of the model may improve (or may not, depending on whether we actually know enough about the materials and the physics), but at a steep price in understandability and analyzability.

The Newton's cradle example shows that physical processes that include instantaneous events are better modeled using functions of the form *p*: ℝ ×ℝ→ ℝ, where ℝ represents the natural numbers, rather than the more conventional *p*: ℝ × ℝ→ ℝ. The latter is adequate for continuous processes, but not for discrete events. At any time *t* ∈ ℝ, the signal *p* has a sequence of values, ordered by their microsteps. This signal cannot be misinterpreted as a rapidly varying continuous signal.

Superdense time is ordered lexicographically (like a dictionary), which means that (*t*_1_, *n*_1_) < (*t*_2_, *n*_2_) if either *t*_1_ < *t*_2_, or *t*_1_ = *t*_2_ and *n*_1_ < *n*_2_. Thus, an event is considered to occur before another if its model time is less or, if the model times are the same, if its microstep is lower. Time stamps are a particular realization of tags in the tagged-signal model of [4].

Note that an alternative model of time that can accomplish the same objectives as superdense time is studied in [43–45]. Their construction is based on nonstandard analysis [46], which, similarly to superdense time, has an infinite number of points at every real time point. These points are represented as convergent sequences, and a total order is induced over these sequences by means of a measure-theoretic construction. It has the property that every non-standard time has an immediate predecessor and an immediate successor, which the authors say provides an operational semantics. However, while an operational semantics does require the notion of a discrete step of computation, it also, requires that the number of steps preceding any given step be finite. That is not automatically provided by the nonstandard semantics, and when it is provided, the solutions seem to be isomorphic with our much simpler superdense time construction. Hence, it does not appear to this author that anything is gained by going into a more complex mathematical formulation.

### 5.4. Numeric Representation of Time

Computers cannot perfectly represent real numbers, so a time stamp of the form (*t*, *n*) ∈ ℝ × ℝ is not realizable. Many software systems approximate a time t using a double-precision floating point number. However, as I noted above, this is not a good choice.

Ptolemy II [47] solves this problem by making the time resolution a single, global constant. Time in a model is given as *t* = *mr*, where *m* is an arbitrarily large integer and the time resolution *r* is a double-precision floating point number. The multiple *m* is realized as a Java BigIntegerThe time resolution *r*, a double, is a parameter shared by all parts of a model. A model, therefore, has the same time resolution throughout its hierarchy and throughout its execution, no matter how big time gets. Moreover, addition and subtraction of time values do not suffer from quantization errors.

In Ptolemy II, the microstep *n* in a time stamp (*t*, *n*) is represented as a 32-bit integer. The microstep, therefore, is vulnerable to overflow. This vulnerability could be avoided by also using a BigInteger, but experience indicates that only defective models exhibit such overflow, specifically models with Zeno conditions that prevent time from advancing. Hence, the additional cost would not be justified. Similarly, the use of a BigInteger to represent *m* may not be justified if overflow is demonstrably preventable.

The Ptolemy II implementation of time illustrates that a rigorous model of time satisfying all of the requirements in Section 5.1 is realizable and practical. In addition, this model of time is superdense and multiform, giving us considerable modeling power.

## 6. Distributed Timed Systems

Models of time can exist purely in the cyber world. For example, synchronous languages [5] have a semantic notion of a global “clock,” but this clock need not have any connection with any physical notion of time. To model cyber-physical systems, a connection needs to be established. What should that connection be? Discrete-event simulators [48–50] and continuous system simulators [51] establish a semantic notion of time that is meant to simulate Newtonian time. However, is Newtonian time the right choice? For CPS, we need a model of time with explicit concern for bidirectional model fidelity; i.e., can we create a physical system for which the model has high fidelity?

The problem gets more challenging as the systems of interest become physically bigger. At the scale of a microchip, for example, engineers know how to maintain a quantized global notion of time with sub-nanosecond precision through clock distribution networks. However, this becomes much harder at the scale of a vehicle or a factory floor and even harder at the scale of a city or a continent. There seems to be a tradeoff between precision and scale. This tradeoff, however, is being challenged today by network clock synchronization protocols, which can deliver astonishingly precisely synchronized clocks over large geographic areas.

In this section, I first review the state-of-the-art in clock synchronization. I then show how this technology can be used to create deterministic CPS models at any scale. There are still tradeoffs, of course, but they are not as simple as precision *vs.* scale.

### 6.1. Clock Synchronization

Einstein firmly rejected Newton's notion of absolute time and established that a physical notion of time is nothing more than a clock. The accuracy of a clock is only meaningful with respect to other clocks. We can accept this and still create deterministic models that use (quantized) Newtonian time and for which there are faithful physical realizations. The models will have faithful realizations only under some assumptions, for example that the motion of the clocks relative to one another is slow compared to the speed of light. However, model fidelity always requires some assumptions and is always approximate. Every model is unfaithful to the thing being modeled outside some assumed regime of operation.

Clock synchronization has a long and distinguished history. The rotation of the Earth and its orbit around the Sun provide a primal clock for humans, one that has always served as a key reference for man-made clocks. An early example of synchronized man-made clocks is the Gregorian calendar. Such a calendar measures the passage of time with a precision of one day, synchronized globally.

Mechanical clocks came of age later, driven by navigation and by the railroads. In the 19th century, railroads even used clock distribution networks, so mechanical clocks ticked in unison. At that time, clock synchronization with a precision on the order of seconds at a continental scale was achieved. This is about five orders of magnitude better than the Gregorian calendar.

In the late 20th century, and continuing today, we are experiencing the next major improvement in clock synchronization. The GPS system provides a globally-accessible clock where the concurrence of two clock readings anywhere on the planet has a precision on the order of about 100 ns. This is about seven orders of magnitude better than what the railroads achieved. The GPS system uses atomic clocks on satellites, triangulation to determine position and relativity to correct for gravitation and satellite motion. GPS, however, is not always available to systems (particularly indoor systems), and it is vulnerable to spoofing and jamming.

Complementing GPS, network clock synchronization began around 1985 with the introduction of NTP (the network time protocol) [52]. The goal was to synchronize computer clocks to UTC (coordinated universal time), which itself is synchronized to the primal clock, the rotation of the Earth. NTP represents time using two 32-bit integers, one counting seconds, one counting fractions of a second (2^−32^ s). Except for the possibility of overflow, this representation of time satisfies Requirements 1–4 of Section 5.1. In practice, NTP synchronizes clocks on the Internet to precisions on the order of tens of milliseconds.

A more recent clock synchronization protocol, standardized as IEEE 1588 [53], can deliver real-time clock precision on the order of a few nanoseconds on a local area network, with some careful design of the network and with a modest amount of hardware support in the Ethernet physical interface circuit (called the PHY). A variant of this protocol forms part of the IEEE 802.1AS family of standards for Ethernet, commonly known as AVB (audio video bridging) and TSN (time-sensitive networking). An illustrative CPS application of IEEE 1588 is the Meyer Sound CAL (column array loudspeaker), which uses synchronized clocks on an Ethernet LAN to coordinate many loudspeakers to do acoustic beamforming. This is used to control the acoustics in large public spaces, such a stadiums.

The accuracy of NTP and IEEE 1588 does not depend on communication delay, but rather depends on the asymmetry of the communication delays. That is, if the latency of communication from point *A* to point *B* is exactly the same as the latency of the communication from point *B* to point *A*, then perfect clock synchronization is theoretically possible. In practice, such protocols can come quite close to this theoretical limit over practical networks. The White Rabbit project at CERN, for example, claims to be able to synchronize clocks on a network spanning several kilometers to under 100 ps [54]. This means that if you simultaneously ask two clocks separated by, say, 10 km of networking cable, what time it is, their response will differ by less than 100 ps. With such precision, even making the measurements that justify this claim is extremely tricky.

With such precision time protocols (PTP), one or several master clocks are elected (and reelected in the event of failure), and slaves synchronize their clocks to the master by messages sent over the network. This guarantees a common notion of time across all platforms, with a well-defined error margin.

A time value in IEEE 1588 is represented using two integers, a 32-bit integer that counts nanoseconds and a 48-bit integer that counts seconds. These numbers are set relative to the PTP epoch, where time zero is 00:00:00 on 1 January 1970 on the TAI (International Atomic Time) time scale. This representation also satisfies Requirements 1–4 of Section 5.1, and it is less vulnerable to overflow than NTP (because of the use of 48 bits instead of 32 for seconds).

Clock synchronization is starting to become common on wireless networks, as well. In the 802.15.4 family of standards (of which ZigBee is one of the better known variants), a mechanism known as TSMP (time synchronized mesh protocol), developed by Dust Networks, is used to coordinate the wake-up times of battery-powered radios. Synchronizing the clocks enables these devices to leave their radios powered off most of the time, which dramatically extends battery life.

I believe that high-precision clock synchronization will be ubiquitous within a few years. Every device on the Internet will have a clock that is synchronized to every other device on the network to within microseconds. This is not achievable with NTP alone, which is an end-to-end protocol that requires no support from the network. To achieve such precise global synchrony will require support in the network, where PTPs are deployed in all switching and transport devices and timing services are provided to the endpoints.

If this comes to pass, the effects will be dramatic. Synchronized clocks enable coordination without communication. They could enable much more efficient use of networking bandwidth, with more deterministic latencies and fewer packet losses. They enable much better energy efficiency, particularly for wireless devices, because wake-up times can be coordinated. They also enable more sophisticated scientific experiments and instrumentation. Additionally, perhaps most importantly, synchronized clocks enable deterministic CPS models with faithful realizations, as I will explain in the next section.

As with any network service, security is a concern. Once devices and services have come to rely on synchronized clocks, an attack on a global clock synchronization protocol could have devastating consequences. However, I believe that synchronized clocks will offer new mechanisms for improving security, primarily because of the key property that they enable coordination without communication. For example, with synchronized clocks, the absence of a message conveys information. Furthermore, with synchronized clocks, it becomes possible to detect deviations from normal behavior that are not detectable otherwise. A denial of service (DoS) attack, for example, is fundamentally a disruption of the timing properties of a network. However, if the network has no temporal semantics, then there is no clear definition of such an attack.

### 6.2. Ptides: A Deterministic CPS Modeling Paradigm

The Ptides (the name comes from the somewhat tortured acronym for “programming temporally-integrated distributed embedded systems;” the initial “P” is silent, so the name is pronounced “tides”) project at Berkeley set out around 2007 to develop a deterministic modeling paradigm suitable for CPS applications at any scale. As with any model, a Ptides model is faithful to a physical realization only under certain assumptions about the physical realization. Specifically, for a Ptides model to be faithful, the physical realization must satisfy the following assumptions:

1. Clocks are synchronized with a known bound on the synchronization error.
2. Every communication channel has a known bound on its latency.
3. The time taken by any computation that may affect the physical world has a known bound.

The second and third assumptions are common in real-time systems, but the first is less common.

As with any model, violations of these assumptions are always possible in any physical realization. Such violations should be treated as faults. The physical system has failed to deliver a faithful realization of the model.

These three assumptions require that bounds be known, not that bounds be small. The first assumption, for example, is conservatively met by NTP over the open Internet if we assume a bound of, say, 10 s and we assume that each physical clock in the system is reasonably stable. With more carefully designed networks and clocks, however, we can get much smaller bounds. With White Rabbit, for example, a reasonable bound to assume might be one nanosecond.

The second assumption requires very pessimistic bounds if messages are transported on the open Internet, but on a more controlled LAN, less conservative bounds are practical. Very tight bounds are possible if we regulate the use of the network using, for example, the prioritized routing of AVB. Even over ordinary Ethernet with TCP/IP, tight bounds are possible if we can prevent packet drops, for example using clock synchronization and schedules to ensure that buffers never overflow in the routers.

The third assumption is reliably met by programs with bounded loops on ordinary microprocessors if we make conservative assumptions about the execution (e.g., bounded or zero interrupts, all memory accesses are cache misses, *etc.*). However, much tighter bounds become possible using a PRET machine.

A Ptides model is deterministic, and whatever bounds we assume, if those bounds are met by the physical realization, then the physical realization will be faithful to the model. The interactions between the cyber and the physical worlds will have a tolerance on timing equal to the assumed bound on the clock synchronization error. Additionally, the order of all actions, globally, will be deterministic. Hence, Ptides provides a credible deterministic CPS modeling paradigm.

#### 6.2.1. Background of Ptides

The Ptides programming model was originally developed by Zhao as part of her Ph.D. research [55,56]. The case for a time-centric approach like Ptides is elaborated in [57], and an overview of Ptides and an application to power-plant control is given by Eidson *et al.* [58]. A more tutorial exposition is given in [38].

A number of implementations followed Zhao's initial work. A simulator is described by Derler *et al.* [59] and an execution policy suitable for implementation in embedded software systems in [60,61]. Zou [62] developed PtidyOS, a lightweight microkernel implementing Ptides on embedded computers, and a code generator producing embedded C programs from models. Matic *et al.* [63] adapted PtidyOS and the code generator to demonstrate their use in smart grid technologies. Feng and Lee [64] extended Ptides with incremental checkpointing to provide a measure of fault tolerance. They showed conditions under which rollback can recover from errors, observing that the key constraint in Ptides is that actuator actions cannot be rolled back. Ptides has also been used to coordinate real-time components written in Java [65].

A technique similar to Ptides was independently developed at Google for managing distributed databases in the Google Spanner system [66]. In this work, clocks are synchronized across data centers, and messages sent between data centers are time stamped. The technique provides a measure of determinacy and consistency in database accesses and updates.

The Ptides modeling paradigm is a variant of a discrete-event (DE) modeling paradigm [48–50]. In a DE model, components interact by sending each other time-stamped messages (called events). A key property of DE is that every component processes events in time-stamp order. This property underlies the determinism of the model.

Variants of DE are widely used in simulation languages, such as DEVS (discrete event system specification) [67]. DE also forms the foundation for widely-used hardware description languages VHDL, Verilog and SystemC. DE is also used in network simulation tools, such as OPNET Modeler (OPNET Technologies, Inc., Bethesda, MD, USA) and ns-3. The SysML implementation in IBM Rational's Rhapsody tool is also a variant of a DE model.

Unlike any preceding DE modeling paradigm, Ptides uses superdense time and multiform time and therefore meets all of the requirements and desirable features in Section 5.1. Moreover, Ptides uses DE not for simulation, but rather for the design of deployable systems.

#### 6.2.2. Structure of a Ptides Model

**Figure 2.**
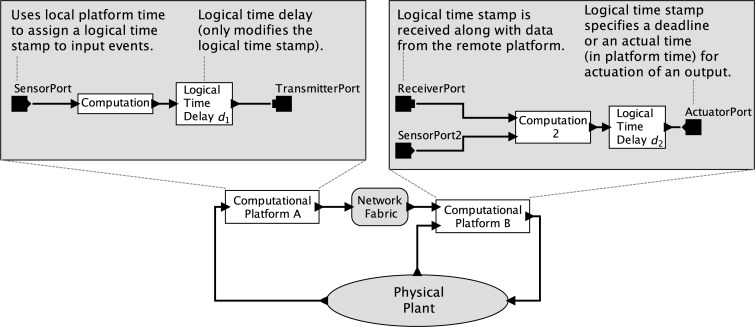
Structure of a simple CPS Ptides model.

Figure 2 elaborates Figure 1, making it a Ptides model. In a Ptides model, sensors and actuators connect the cyber and the physical worlds. On the physical world side, the model uses idealized Newtonian time with a tolerance equal to the bound on clock synchronization error. On the cyber side, each platform that performs computation is assumed to have a clock that is synchronized (with bounded error) with all of the other clocks in the system.

When a sensor takes a measurement, the measurement becomes an event, with its time stamp being the local platform time at which the measurement is taken. In Figure 2, there are two sensors, which, in the Ptides model, are represented as SensorPortand SensorPort2. These are components in the model that produce time-stamped events. Since these components are on different platforms, their time stamps will be taken from different local clocks.

For actuation, the software delivers a time-stamped message to an actuator. In Figure 2, there is one actuator, represented in the model as ActuatorPort. The time stamp of an event delivered to an actuator can be interpreted as a deadline, meaning that the event should be delivered to the actuator before the local platform time matches the time stamp. The actuator can then either immediately perform the actuation or delay the actuation until the local platform time matches the time stamp. The latter strategy is usually preferred, because it delivers deterministic cyber-physical interactions. Up to the time tolerance, the order of actuations in the physical world is defined.

In between sensors and actuators, there may be computation and network communication. Network communication is treated similarly to sensing and actuation, but with a slight difference in how time stamps are handled. First, time stamps are carried through the network along with any message payload. In Figure 2, the TransmitterPort will bundle the payload together with a time stamp and send it over the network to the ReceiverPort. The ReceiverPort will unbundle these, using the received time stamp for the event it produces on platform *B*. What is that time stamp?

Notice that platform *A* will send a message over the network in response to receiving sensor data on SensorPort. Suppose the sensor data has time stamp *τ*, the local platform time at which the sensor measurement is taken. The sensor data will be processed by the box labeled Computation. Assume that that box produces an output event with the same time stamp *τ*. The second box on platform *A* is labeled “Logical Time Delay *d*_1_.” What that box does is simply pass its input event to its output, but with the time stamp *τ* replaced with time stamp *τ + d*_1_. Hence, the ReceiverPort will receive a message with time stamp *τ* + *d*_1_. When will it receive the message?

The time stamp of an event delivered to a network transmitter port is treated as a deadline (technically, this deadline can be relaxed by introducing a known offset, but the logic is easier to understand if we assume that the offset is zero, as I do here), just as if the transmitter port were an actuator. Like an actuator, the implementation may send the message as soon as possible or it may delay sending the message until the local platform time matches the time stamp. The latter behavior can be used to regulate the use of the network, for example to avoid media collisions or buffer overflows.

On platform *B*, an interesting thing happens at the box labeled “Computation 2.” Here, there are two converging streams of time-stamped events. Since Ptides follows DE semantics, these events must be processed by Computation 2 in time-stamp order. If two messages have numerically identical time stamps, then Computation 2 must see them simultaneously. How can we accomplish this?

Suppose that SensorPort2 makes a measurement and produces an event with time stamp *t*. When can Computation 2 process that event? In order for Computation 2 to process the event, we must ensure that ReceiverPort will not later produce an event with time stamp *t* or less. How can we be sure of this?

Here, Ptides uses Assumptions 1 and 2 above. Assume the bound on the clock synchronization error is *e* and the bound on the network communication latency is *d*. Then, Computation 2 can safely process an event with time stamp *t* when the local platform clock reaches or exceeds time *t* + *d* + *e*. Why is this safe?

Suppose that the event sent from platform *A* to *B* has time stamp *τ* + *d*_1_ < *t*. Because the network has bounded latency, that message will be received by platform *B* before time *τ* + *d*_1_ + *d* < *t* + *d*. However, that time is with respect to platform *A*'s clock, not platform *B*'s clock. Since the error between these clocks is no more than e, the message will be received before time *τ* + *d*_1_ + *d* + *e* < *t* + *d* + *e* on platform *B*. Thus, if no such message has been received by time *t* + *d* + *e*, it is safe to process the event with time stamp *t*. This is coordination without communication!This safe to process analysis is central to Ptides.

Once Computation 2 has executed, producing an event on its output with time stamp *t*, local time already exceeds *t* by at least *d* + *e*. Hence, we require another logical time delay component to modify the time stamp to *t* + *d*_2_, where *d*_2_ > *d* + *e*, before delivery to the ActuatorPort. Otherwise, the deadline would have already elapsed. While it is clearly necessary for *d*_2_ > *d* + *e*, is it sufficient? What if Computation 2 takes a long time to complete?

With the assumed bounds on clock synchronization error and network latency, a correct implementation of Ptides is deterministic in that a sequence of time-stamped events from sensors always results in a unique and well-defined sequence of time-stamped events delivered to actuators. The safe-to-process analysis guarantees this. However, this determinism does not provide any guarantee that events are delivered to actuators on time (prior to the deadline given by the time stamp). The problem of determining whether events can be delivered on time to actuators and network transmitter ports is called the schedulability problem. The question is, given a Ptides model like that in Figure 2, does there exist a schedule of the firing of components, such that deadlines are met on a particular execution platform? Like all real-time systems problems, this question cannot be answered unless we can bound the execution time of software components, and such bounds are hard to come by [23]. Hence, we need Assumption 3 above, that execution times can be bounded. PRET machines make it easier to construct these bounds, but even if we can bound the execution time of each software component, the question of whether there exists a scheduling policy that meets the deadlines remains. Zhao solved this problem for a limited class of models [56]. The problem is further discussed by [61] and largely solved in [68].

Hence, if a Ptides model is schedulable on a particularly physical realization and if the physical realization delivers bounded clock synchronization error and bounded communication latencies, then the physical realization will be faithful to the deterministic CPS model. All of the assumptions are achievable, and therefore, we have an existence proof for deterministic CPS models with faithful physical realizations.

The most interesting, subtle and potentially confusing part about Ptides is the relationship between multiple time lines. The model of the physical environment uses a (quantized) Newtonian ideal time. Each platform contains a clock that progresses at its own rate (having a multiform relationship to Newtonian time), but periodically corrects its rate to remain synchronized with other clocks. This gives *N* platform time lines, one for each of *N* platforms. Overlaid over this is another time line, the logical time line of the time stamps. Time stamps are digital numbers in some format (e.g., IEEE 1588 or Ptolemy's BigIntegers), where order and simultaneity are clearly defined. Every correct execution of a Ptides model ensures that every component processes events in time-stamp order. However, reasoning about these systems takes considerable practice, since most of us are not at all used to reasoning about multiple time lines simultaneously. Fortunately, CPS designers can focus exclusively on the logical time of time stamps, leaving the other timelines for the compiler to deal with.

#### 6.2.3. Comparison with Parallel DE Simulation

Since Ptides provides distributed execution of a DE model, it provides a solution to the problem of executing DE models in parallel. There is a long history of work on parallel execution of DE models [69–72].

The classical Chandy and Misra approach [69] offers an alternative to the safe-to-process analysis of Ptides. In this approach, to process the sensor event with time stamp t at Computation 2 in Figure 2, we would wait until we receive an event from the network channel and then process whichever event has the lesser time stamp. However, this could result in quite a wait, particularly if a fault occurs and the source of events on this path fails. For the purposes of simulation, the Chandy and Misra approach may be sensible, because if a component fails, the whole simulation becomes suspect. However, in a deployment, this would make the system far too brittle. Any failure anywhere in a distributed system could bring the whole system down.

An alternative approach due to Jefferson [71] would process the event with time stamp t speculatively, assuming no problematic event will later arrive. If an event later arrives with a time stamp less than t, then this “time warp” approach reverses the computation by restoring the state of the system to what it was before processing the event. For CPS applications, this approach is fundamentally limited by the inability to backtrack actuators [64].

#### 6.2.4. Fault Handling

Because no physical realization is perfectly faithful to any deterministic model, a Ptides execution may find itself in a situation where it has processed an event with time stamp *t*, and it later sees an event with a lesser time stamp. In this case, we know that one of our assumptions was violated. It is not possible to tell which assumption was violated, however. Violating the network delay bound, for example, is indistinguishable from violating the clock synchronization error bound. Nevertheless, the violation is detectable, so we can treat this as a fault condition. A well-designed CPS application based on Ptides models will include a fault handling strategy to deal with such scenarios. In Google Spanner, for example, a database transaction mechanism is overlaid on top of the Ptides mechanism, rejecting a transaction is the fault handling strategy.

Once we have fault handling, we can trade off the conservatism of our assumptions against the frequency of faults. If we aggressively assume a low latency bound on communication, for example, then we will experience more faults than with a more conservative assumption. Not all faults are serious, so the ability to explore such tradeoffs systematically can be a very valuable design tool.

As long as faults do not occur, the execution will look exactly the same as the physical environment, up to the time tolerance. This makes Ptides models much more robust than typical real-time software, because small changes in the (physical) execution timing of internal cyber events are not visible to the physical environment (as long as real-time constraints are met at the sensors, actuators and network interfaces). Moreover, since the execution of a Ptides model carries time stamps at run time, run time violations of deadlines at actuators can be detected. Ptides models can be easily made adaptive, changing modes of operation, for example, when such real-time violations occur. In general, therefore, Ptides models provide adequate runtime information for detecting and reacting to a rich variety of timing faults.

## 7. Conclusions

This paper is about better engineering of cyber-physical systems through better models. I have emphasized that models must not be conflated with the thing being modeled, an altogether too common error among engineers. I have argued that deterministic models have historically proven extremely useful and even form the kingpin of the industrial revolution and the digital and information technology revolutions. Key deterministic models include differential equations, synchronous digital logic, single-threaded imperative programs and instruction set architectures. Cyber-physical systems, however, combine these models in such a way that determinism is not preserved. I then describe two projects that show that deterministic CPS models with faithful physical realizations are possible and practical. The first project is PRET, which shows that the timing precision of synchronous digital logic can be practically made available at the software level of abstraction. The second project is Ptides, which shows that deterministic models for distributed cyber-physical systems have practical faithful realizations. Ptides leverages network clock synchronization, which I believe will become ubiquitous. These projects are mere existence proofs, and there is no doubt that more work is needed before engineers routinely use deterministic models for CPS. I conclude by speculating that a good deterministic modeling paradigm for CPS will fuel the next technology revolution.
